# The impact of silver nanoparticles on the growth of plants: The agriculture applications

**DOI:** 10.1016/j.heliyon.2023.e16928

**Published:** 2023-06-02

**Authors:** Sajad Khan, Muhammad Zahoor, Raham Sher Khan, Muhammad Ikram, Noor Ul Islam

**Affiliations:** a Center for Biotechnology and Microbiology Abdul Wali Khan University Mardan, Mardan, 23200, Khyber Pakhtunkhwa, Pakistan; b Department of Biochemistry, University of Malakand at Chakdara, Dir Lower, Khyber Pakhtunkhwa, Pakistan; c Department of Chemistry, Abdul Wali Khan University Mardan, Mardan, 23200, Khyber Pakhtunkhwa, Pakistan; d Department of Chemistry, University of Malakand at Chakdara, Dir Lower, Khyber Pakhtunkhwa, Pakistan

**Keywords:** Crops protection, Food packaging, Nanotechnology, Seeds germination, Silver nanoparticles

## Abstract

Nanotechnology is the most advanced and rapidly progressing field of science and technology. It primarily deals with developing novelty in nanomaterials by understanding and controlling matter at the nanoscale level. Silver nanoparticles (AgNPs) are the most prominent nanoparticles incorporated with wide-ranging applications, owing to their distinct characteristics. Different methods have been employed for nanoparticles synthesis like chemical method, physical method, photochemical method, top-down/bottom-up approach and biological methods. The positive impacts of silver nanoparticles have been observed in various economy-based sectors, including agriculture. The scientific curiosity about AgNPs in agriculture and plant biotechnology has shown optimum efficacy over the last few years. It not only enhances seed germination and plant growth, but also improves the quantum efficiency of the photosynthetic process. AgNPs play a vital role in agriculture by having several applications that are crucial for ensuring food security and improving crop production. Moreover, they also act as nano-pesticides, providing sufficient dose to the target plants without releasing unnecessary pesticides into the environment. Nano-fertilizers slowly release nutrients to the plants, thereby preventing excessive nutrient loss. AgNPs are utilized for effective and non-toxic pest management, making them an excellent tool for combating pests safely. They combine either edible or non-biodegradable polymers for active food packaging. In addition, AgNPs also possess diverse biological properties such as antiviral, antibacterial and antifungal activities, which protect plants from hazardous microbes. The aim of this review is to comprehensively survey and summarize recent literature regarding the positive and negative impacts of AgNPs on plant growth, as well as their agricultural applications.

## Graphical abstract

**Figure:**
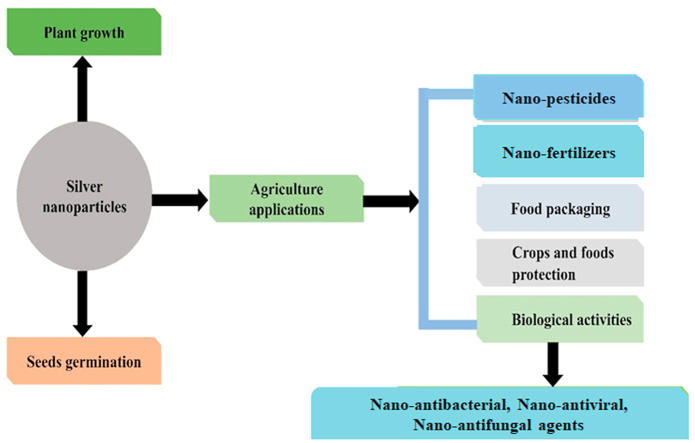

## 1 Introduction

Nanotechnology is an emerging field of science that mainly deals with nanomaterials to overcome size limitations and change the world perspective on science. It has revolutionized agriculture sectors and played essential roles in various fields like agricultural biotechnology, food security, and crop production. Nanoparticles distinct physicochemical features are extremely helpful in inducing plant metabolism. However, their interactions with plants have not been clarified and understood in detail. Several contradicting reports of nanoparticles regarding accumulation, translocation, absorption, biotransformation, and toxicity were reported in diverse plant species. AgNPs are one of the most significant nanomaterials whose impacts are still under investigation [1,2]. The synthesis of plant-based nanoparticles is an excellent approach due to their low cost, non-pathogenic factor, non-toxic phytochemical constituents, flexibility in reaction parameters, and biochemical diversity of plant extract [3]. Approximately 25% of all nanotechnology products incorporate its usage. Such particles have a large surface area in relation to their volume, and they typically measured between 1 and 100 nm in size [4,5]. AgNPs have distinct characteristics and broad range of applications, particularly in agriculture and plant biotechnology. They have been shown to enhance seed germination, plant growth, and photosynthetic efficiency, whereas also acting as safe and effective nano-pesticides and fertilizers. The antibacterial, antifungal, and antiviral properties of AgNPs have been widely utilized in various industries, including healthcare, textiles, building materials, medical devices, food services, cosmetics, and household materials. They are beneficial in these industries because of their ability to protect against harmful microorganisms and provide a safer and healthier environment [6,7]. AgNPs acts as a photochemical and good electric conductor, showing wastewater treatment and electronic device implementation. It enhances fruit ripening, plant growth, and fungicides in agriculture fields [8,9]. In general, fertilizers are essential to increase the growth and development of plants. Unfortunately, most fertilizers cannot reach plant bodies due to several factors, such as degradation by photolysis, decomposition, hydrolysis, and leaching. Thus, it is essential to reduce the loss of nutrient in fertilization and improve crop yield by manipulating novel nanomaterials and nanotechnology applications. Nano-fertilizers, also known as nano-encapsulated nutrients, have several properties, including effectiveness for crops, controlling the release of chemical fertilizers, releasing the nutrients on demand, regulating plant growth, and enhancing target activity [10,11]. Recently, nanoparticles have attracted the attention and interest in the agricultural applications that effectively minimizes the use of chemical fertilizers and enhances crop growth and yield. Metal nanoparticle phytotoxicity for plant species has recently been investigated in seed germination and root elongation tests to advocate its use in agricultural applications [12,13].

Recent studies showed that plant response to AgNPs is associated with their dosage, which can either enhance or inhibit growth. Exposure to specific and serial AgNPs enhances plant growth more than control plants, whereas low and high concentrations negatively affect plant growth [14,15]. Several reports suggest that proper concentrations of AgNPs are vital in enhancing plant growth/seed germination, improving chlorophyll content/photosynthetic efficiency, and increasing fertilizer and water efficiency [16,17].

## 2 Methods used to synthesize nanoparticles

There are different approaches (methods) used to synthesize nanoparticles which are discussed below.

### 2.1 Chemical method

The chemical method of nanoparticle synthesis is the most convenient, effective, and easy to handle. The chemical reduction technique involves the utilization of both organic and inorganic reducing agents, is the most commonly used technique for synthesizing nanoparticles. Generally, diverse types of reducing agents are typically employed for reducing silver ions (Ag+) in both aqueous and non-aqueous solutions that include: ascorbate, polyol process, sodium borohydride (NaBH_4_), sodium citrate, poly (ethylene glycol)-block copolymers, N-dimethylformamide (DMF), Tollens reagent, N, and elemental hydrogen. Silver nanoparticles react with these agents, reduce Ag^+^ and resulting in metallic silver (Ag^0^). Subsequently, the particles aggregate into oligomeric clusters, ultimately leading to the synthesis of silver particles [18]. Protective agents are used to stabilize dispersive NPs by protecting them during the preparation of metal nanoparticles that can be bind onto or absorbed on surfaces to avoid agglomeration. It can be possible with low-cost with high productivity compared to other methods of NPs synthesis. The chemical approaches of silver nanoparticles in true solution consist of three steps: (i) metal-based precursors, (ii) capping oxidants (iii) reducing agents. To obtain smaller size and uniformly spherical-shaped metal-based nanoparticles, it is essential to employ capping agents that help control the growth of nanoparticles during the synthesis process. The synthesis of a colloidal solution of silver nanoparticles involves two stages of growth and nucleation, which determine the morphology and size of the resulting nanoparticles. Nevertheless, the synthesis of uniformly sized and well-dispersed nanoparticles depends on achieving a consistent growth rate and size during the production process. The synthesis of single dispersed nanoparticles with regular size depends on the production of growth rate and similarity in size. The regulation of the reaction is crucial in measuring the subsequent development of primary nuclei using diverse limits such as temperature, pH, precursors, capping material (PVP), and reducing substance.

### 2.2 Physical method

In this technique, nanoparticles are synthesized by the process of condensation or vaporization by sustaining the furnace tube at atmospheric pressure. The physical synthesis of AgNP is possible in two techniques; evaporation–condensation and laser ablation [19]. The substances present inside the fixed vessel evaporates into the carrier gas in the furnace. In the condensation and vaporization method, nanoparticles of several materials are synthesized such as Zn, Ag, Cu, Pb, and Sn [20]. The synthesis of nanomaterial using a furnace has many drawbacks such as cylinder-shaped particles filling a massive space; a high amount of energy being consumed that causes a huge increment in the environmental heat. In addition, it also requires a prolonged period to achieve thermal constancy. There are many kilowatts of energy needed; thus, to obtain a stable temperature heating period of 10 min is required for the distinctive cylinder-based furnace. Moreover, laser ablation of bulk materials in a solution is an alternative method for synthesizing AgNPs. The laser-based technique is superior compared to the other technique, as no chemical reagent produces in the solution and resulting in pure colloids [21]. Mostly, physical synthesis consumes the physical energy to produce nanoparticles with fine-size circulation. In this type of method, a huge amount of product is fabricated in a single-step procedure and synthesizes a higher number of silver nanoparticles in ash form. When a pulsed laser is directed towards a liquid environment, it only affects the AgNPs that originate from the base metal source, without any involvement of other substances such as ions, compounds, or reducing agents. Several parameters influence the characteristics of synthesize metal NPs such as kind of base metal source, duration of irradiation, laser power, and property of liquid media. Laser ablation synthesize NPs in pure form with no contamination, as it utilizes mild surfactants in the solvent and does not involve chemical reagents [22].

### 2.3 Photochemical method

Photochemical processes have been a primary focus for researchers in the metallic nanoparticles fabrication due to the superior temporal and spatial control [23]. In this method, the solution containing metal precursors exposes to ultraviolet (UV) or visible light. The use of photochemical processes for creating metallic nanoparticles is highly advantageous compared to other methods because it avoids the need for toxic or harmful substances, neither require expensive equipment nor highly skilled personnel, and can be performed under normal ambient conditions i.e., atmospheric pressure and room temperature [24]. The fabrication of metallic nanoparticles in solution utilizes various reagents, including reducing agents, and metal precursors (complex or salt), but rarely use stabilizing agents [25]. The process of photochemistry is employed to begin the reduction of a metal precursor by converting the reducing agent from its n + valence state (Mn^+^) to a zero-valence state (Mn^0^) [26]. The M^0^ forms nuclei or nucleation centres that aggregate and grow to synthesize metallic NPs. Both capping agents and stabilizers play a vital role to control the formation of homogenous metallic NPs with respect to the desire shape and size, avoid agglomeration, and enhance colloidal stability [27]. Polymers trap and protect the NPs against coalescence and oxidation. Furthermore, due to their varying chemical compositions, polymers exhibit specific interactions with the surface of metallic materials, activating vital modification in the size and shape of the resultant nanoparticles. The process of photo-reducing layers of silver nitrate yields AgNPs, which rely on the presence of inert soil suspensions as stabilizing agents to avoid particle aggregation and act as oxidants. The process of irradiation splitting was utilized on silver nanoparticles with a distribution ratio to achieve a constant distribution ratio and absolute diameter size [28].

### 2.4 Top-down and bottom-up approach

The synthesis approaches of metal NPs consist of two approaches namely top-down and bottom-up. The top-down approach involves breaking down bulk materials using physical methods to produce the desired nanostructures (ball milling, ultrasonic machining, laser ablation, etc.). This technique is valuable for its ability to generate a substantial quantity of nanoparticles. However, there is certain limitation of this technique: requires costly equipment and high amount of energy, while the bottom-up approach entails the synthesis of nanoparticles utilizing chemical agents that assembles single molecules and atoms into bigger nanostructures to produce nano-sized materials using biological and chemical approaches. At present, methods for producing synthetic nanoparticles are classified into three main categories: chemical, physical, and biological green synthesis.

### 2.5 Biological methods

In recent times, the biological synthesis of metal nanoparticles has garnered significant attention owing to its broad relevance in diverse fields. It employs biological entities including microorganisms (bacteria or fungi) plant extracts are considered valuable alternatives compared to other synthesis routes.

#### 2.5.1 Microorganisms mediated synthesis

The biosynthetic approach that utilizes natural reducing agents for nanoparticle syntheses such as biological microorganisms (bacteria or fungi), plant extracts, and polysaccharides is also known as green chemistry. Microbes, including bacteria and fungi, remediate the toxic materials by reducing metal ions and synthesizing metal nanoparticles in a biological way [29]. There are certain bacteria that can synthesize AgNPs intracellularly, where intracellular components perform their function as stabilizers and reducers [30]. The naturally available reducing agents that synthesize AgNPs biologically might be a promising technique that replaced the most complex physiochemical process. Green synthesis is superior to other methods due to the absence of toxic chemicals and harmful by-products but employs natural capping agents to stabilize AgNPs [31]. Bacteria generally utilize nitrate as a main source of nitrogen, in which reductase converts the nitrate into nitrite, employing the reducing power of nicotinamide adenine dinucleotide (NADH). The metabolic processes of bacteria reduce nitrate to nitrile and ammonium, which might be utilized in the bio-reduction of Ag^+^ ions by an intracellular electron donor. The enzyme nitrate reductase is an optimum reducer in the process of bio-reduction of silver ions. The green synthesis of AgNPs was performed by another plausible mechanism that involves the biological system of a fungus, *Verticillium species*. The AgNPs synthesis initiates beneath the cell wall surface, rather than the aqueous solution. Fungal cells' surface traps the Ag^+^ ions because of the electrostatic interaction between negatively-charged carboxylate groups of the enzyme and Ag^+^ ions. The formation of Ag nuclei occurs after Ag^+^ ions intracellularly reduce the cell wall, and further expansion occurs after Ag^+^ ions are reduced. Bacteria are highly capable of producing inorganic components both inside and outside the cell. As a result, they can serve as highly effective bio-factories for synthesizing nanoparticles e.g., gold, copper, silver, etc. The synthesis of silver nanoparticles is a well-known example of biologically derived nanoparticles that possess biotic characteristics.

#### 2.5.2 Green routes for nanoparticle synthesis

Despite being in its early stages, research in the field of nanotechnology for environmentally friendly production of nanoparticles is expanding rapidly in recent years. The nanoparticles are synthesized either using plant extracts or microorganisms such as algae, bacteria, fungi, or yeast. The incorporation of this approach marks a noteworthy stride towards accomplishing the twelve principles of green chemistry established by Paul Anastas and John Warner in 1998 [32].

The synthesis of nanoparticles using microorganism is generally a highly complex and less cost-effective than utilizing plant extracts. The maintenance of cell cultures and extraction of nanoparticles from microorganisms can be challenging, making the utilization of plant extracts a more appealing option, particularly for large-scale nanoparticle production. Dried grass is generally simpler and less expensive than cultivating bacterial or fungal strains [33].

#### 2.5.3 Plant mediated synthesis of nanoparticles

The process of collecting plants and extracting active biomolecules to synthesize silver nanoparticles is relatively straightforward process. The extract of plants contains several biomolecules, including alkaloids, terpenoids, sugars, NADH-dependent reductase, phenols, flavonoids, proteins, and tannins. Biomolecules possess the capability to reduce metal ions or stabilize particle growth after nucleation. Biomolecules such as enzymes, vitamins, proteins, lipids, and organic acids actively take part in the reduction and stabilization of nanoparticles during NPs synthesis. Generally, the active molecules hold amine, carbonyl, methoxide or hydroxyl functional groups [34].

It should be emphasized that the plant material is not the only factor that influences the production of nanoparticles with specific structures, sizes, or shapes. The conditions under which nanoparticles are synthesized, such as the pH level, exposure to sunlight, temperature, duration of mechanical stirring, and dispersion, can all have an impact on the resulting nanoparticle's size, structure, and chemical properties. One of the earliest approaches for producing metallic nanoparticles using plants involved utilized alfalfa sprouts. This method represents the first documented instance of synthesizing silver nanoparticles (AgNPs) using a living plant system. The roots of Alfalfa possess the distinctive property to assimilate Ag from agar medium and subsequently transported it to plant shoots in an identical oxidation state. After being transported to the plant shoots, the Ag atoms begin to self-organize and form nanoparticles by bonding with one another and forming a larger arrangement. When it comes to green synthesis, plants seem to be a more efficient and speedy option compared to fungi and bacteria. Initial investigations have demonstrated that these synthesis procedures are capable of rapidly producing Ag NPs. It involved extracts and plant metabolites, as well as products of biological macromolecules: lipids, carbohydrates, proteins or peptides, and nucleic acids. It is now well-proven that nanoparticle synthesis on the base of biological route saves energy and creates comparatively less harmful waste, making it a promising alternative to conventional synthesis methods. Green nanotechnology has become a driving force for both academic and industrial research, promoting the design and development of Green Nanoparticles (GNPs) with specific applications. GNPs have already been implemented in various innovative technologies, including smart electronic devices, life-saving nano-pharmaceuticals, and environmentally friendly energy production devices. This field not only fosters fundamental research but also emphasizes the importance of goal-oriented research for practical applications. Plant-based nanoparticles are tiny particles that are created from plant materials. These nanoparticles are attracting attention due to significant applications in diverse fields: medicine, agriculture, and environmental protection. The process of synthesizing these particles involves extracting active components from diverse plants part, including stems, leaves, seeds, and roots, and then treating them with various chemical or physical methods, including reduction, oxidation, and thermal treatment, to form nanoparticles. There are different methods to make plant-based nanoparticles, including bio-reduction, template-assisted synthesis, and sol-gel methods. Bio-reduction involves using plant extracts to reduce metal ions and form nanoparticles, while template-assisted synthesis uses plants as templates to grow the particles. Sol-gel methods use plant-based precursors to form nanoparticles through gelation. Compared to synthetic nanoparticles, plant-based nanoparticles have many benefits, including biocompatibility, biodegradability, and low toxicity. They can also be easily modified with biological molecules, making them useful for medical applications. The plant-based nanoparticle synthesis is a promising research area with many potential applications. With the development of science, researchers have focused on the need of developing efficient techniques that synthesized environmentally friendly and non-toxic biocompatible nanoparticles [35]. Plants harbor several bioactive chemicals such as Flavonoids, aldehydes, phenols, amino-acids, vitamins and ketones, that are present in plants, which can reduce valuable metal ions [36]. Plant-mediated synthesis is recognized as a less costly, environmentally-friendly, and easily up-scalable method of synthesizing nanoparticles. Due to their widespread availability in different climates, plant extracts are easily accessible and economical for the nanoparticles production. One of the key benefits of using plant extracts for the production of nanoparticles is the availability and ease of access to precursor materials, which can be a major benefit in the synthesis process.

Although plant extracts are a convenient source for nanoparticle synthesis, it can be more difficult to control the shape, size, and composition of the resulting nanoparticles compared to chemical methods. This is because plant extracts consist of complex mixture of organic molecules, which can make the synthesis process more challenging. Despite the difficulty in controlling nanoparticle characteristics when using plant extracts, one advantage is the ability to coat the external surface of nanoparticles with active plant molecules, allowing for functionalization. The combination of nanoparticles and plant molecules creates a synergistic effect that results from the interaction between the intrinsic properties of the nanoparticles and the specific properties of the plant molecules. This synergistic effect is particularly important in the plant-based synthesis of nanoparticles used as antimicrobials [37].

#### 2.5.4 Algae mediated synthesis

Marine algae are a well-known functional food source that is rich in various biologically-active substances, such as minerals, vitamins, lipids, proteins, and polysaccharides, due to their enrichment with these nutrients. The medicinal potential of algae is high against allergy, oxidative stress, cancer, hypertension, lipidemia, thrombosis, and other degenerative diseases [38]. Sponges and diatoms are one of important biological entities present in marine resources. It is a nanostructure consisting of covering coral reefs and silica. Hence, algae also act as “bio-factories” in the production of silver nanoparticles. Algae are valuable compared to different types of bio-reductants due to cost-effectiveness, strong capacity of metal uptake, and macroscopic structure [39].

The novelty of this review lies in its comprehensive coverage of the multiple uses of AgNPs in agriculture and plant biotechnology, which are vital for ensuring food security and improving crop production, while also being safe and non-toxic for the environment. Additionally, it highlights the antiviral, antibacterial, and antifungal properties of these nanoparticles that protect plants from hazardous microbes, making them a promising tool for sustainable agriculture.

## 3 The impacts of silver nanoparticles (AgNPs) on germination of seed

The applications of AgNPs have received high focus and promotion in the medical and pharmaceutical fields. However, the utilization of AgNPs in the agricultural sector is a new research area. In contrast to AgNPs toxicity and anti-microbial properties, recent studies have explored their role in enhancing seed germination and improvement of crops. Despite remarkable significance in the agriculture field and positive effects on seed germination, their applications have also been associated with negative effects.

### 3.1 Positive effects on seed germination

Seed germination is one of the most vital phase for establishing plants in the agriculture and is essential for the quality of crops [40]. AgNPs can incorporate with seed coats and helps in water uptake inside seeds, promoting starch metabolism and seed germination. It is also applied as a nano-priming agent to enhance rice-aged seeds and starch metabolism. One of the priming aims of AgNPs is to regulate up the expression of aquaporin genes, thus encouraging the diffusion of H_2_O_2_ and water. AgNPs was synthesized through a green route using kaffir lime leaf extract as a nano priming agent to enhance rice-aged seed germination. The priming of rice-aged seeds with photosynthesized AgNPs (5 and 10 ppm) has remarkably enhanced the performance of seedling vigor and germination to conventional hydropriming, AgNO_3_ priming, and unprimed control. Nano priming is also associated with strengthening α-amylase activity and thus causes higher soluble sugar content to support seedlings' growth [41]. The extracellular AgNPs were synthesized using *Bacillus subtilis spizizenni.* Bajra (*Pennisetum glaucum)* seeds were treated with 1 mM AgNPs and found excellent for better seed germination in just 3 days. The statistical analysis by one-way analysis of variance showed a vital improvement in plumule and radicle length than control seeds of Bajra [42]. In a study, AgNPs were applied to check salinity stress in lentil seeds. Seedling growth and Lentil seed's seed germination were enhanced after exposure to AgNPs. The germination percentage was also increased for lentils at concentrations of 10 μg/mL AgNPs, enhanced the mean germination time for lentil plants, improved the germination parameters and resulted in better tolerance under drought conditions. AgNPs applied at a concentration of 10 μg/mL were found to improve lentil seed germination under conditions of drought stress [43]. Gum Arabic coated (Ga-AgNPs-6nm) and PVP-coated Ag nanoparticles (PVP-AgNPs- 20 nm) was applied to the seeds of eleven species in different concentrations such as 1, 10, and 40 mg/L. The application of GA-AgNPs reduced the rate of germination in three species and improved one species, while the application of 40 mg showed no effect on germination [44]. AgNPs were synthesized using leaf extract of *Moringa oliefera* and tested on seed germination and growth. The diverse AgNPs concentrations at 25 ppm, 50 ppm, 75 ppm, and 100 ppm were tested with control group to enhance wheat seed germination and seedling growth. In contrast, AgNPs concentration at 100 ppm displayed tremendous growth compared to the control. Additionally, a significant improvement in dry root weight, fresh root weight, root elongation, and root length were reported at 100 ppm AgNPs concentration [45]. In a study, thirty seeds in each Petri dish were applied with concentrations of silica (20%) and AgNPs (60%) with controlled distill water treatment. Seed germination started on the fourth day and was controlled for 14 days. The results of the analysis showed that AgNPs (20%) yielded higher values in seed germination properties [46]. In another study, AgNPs were prepared at different concentrations such as 0, 10, 20, 30, and 40 μg/mL in which 30 μg/mL significantly enhanced percent germination rate and seed germination due to higher germination potential. AgNPs with size range of 20 nm were diluted to seven concentrations: 0.05, 0.1, 0.5, 1, 1.5, 2, and 2.5 mg/L to check the seeds germination [47]. The treatments of AgNPs have improved the germination rate in watermelon (*Citrullus lanatus*), zucchini (*Cucurbita pepo*) and corn (*Zea mays*) [17,18]. AgNPs were synthesized using glucose (green reagent). AgNPs at concentration of 0.001–0.5 mg/L showed no effect on seed germination, while 0.06–1 mg/L and 0.03–0.1 mg/enhanced root and shoot fresh mass. AgNPs at concentrations ranging from 0.06 to 0.5 mg/L have significant applications in agriculture, including their use as an eco-friendly alternative source of fertilizer for wheat crops [49]. AgNPs and AgNO_3_ were used at 100, 500, and 1000 mg/L; 100 and 500 mg/L, respectively, in *Brassica juncea* var. The toxicity of these particles was evaluated using the Varuna mustard plant. The results indicated that AgNPs caused a minor improvement in the length of root and shoot. It increased chlorophyll as well as protein content at all concentrations compared to the control [50]. In a study, the applications of AgNPs and salt at concentrations of 0, 40, 60, and 80 ppm; 0, 30, 60, 90, and 120-mMol^−1,^ respectively, exhibited that 80 ppm AgNPs effect was excellent (*P* ≤ 0.05) on percentage germination of seed. AgNPs application was essential to enhance the salinity tolerance in the *S. hortensis* seedling. It was crucial in activating various plants defense mechanisms against salt toxicity [51]. The economically vital crop, pearl millet (*P. glaucum* L.), was grown on M.S. basal medium with different concentrations of AgNPs (T1 = control, T2 = 20 ppm, T3 = 40 ppm, T4 = 60 ppm, and T5 = 80 ppm). The biochemical profile, seedling growth, and seed germination of pearl millet were highly affected (p ≤ 0.05) and found excellent in T3 treatment. Compared to the control, the T3 treatment showed the highest seedling vigour index (VI), seed germination, biomass accumulation, and root/shoot length [52]. *Alnus nitida*-based leaves extract was used to synthesize AgNPs, which were then applied to wheat seeds at varying concentrations (0.75 μg/mL, 1.5 μg/mL, 3 μg/mL, 6 μg/mL, and 15 μg/mL) under *in vitro* conditions. The study revealed that the wheat plants treated with 6 μg/mL of AgNPs exhibited significant enhancements in dry and fresh weights, as well as induced the production of secondary metabolites [53].

### 3.2 Negative effects on seed germination

AgNPs have been found to have several beneficial effects on plant growth, certain plants have shown toxicity in response to their application, indicating potential negative effects. The application of AgNPs at different concentrations such as 1000 μg/mL, 1600 μg/mL and 1200 μg/mL on *Brassica campestris, Oryza sativa and Vigna radiata r*espectively. Ions inhibited roots at 4500 μg/mL and 6000 μg/mL concentrations. The roots length was retarded to 1% and 0.5% in *Oryza sativa* and *Brassica campestris*; *Vigna radiata,* respectively [54]. AgNPs at 10–20 mg/L reduced germination percentage in *Lolium perenne* [55]. In a study, three AgNPs (1–20 nm) were used for seed germination. The highest germination was observed after wheat seeds were incubated at 25 °C for 7 days in a dark environment. The result indicated that the AgNPs effect on wheat plants germination was reduced only at 10 mg/L application. At the same time, it was clarified that there was zero effect on other applications [56]. AgNPs (20 nm) exposure at 75 μg/L concentration has not shown any changes but reduced the germination rate of *Arabidopsis thaliana* [57].

## 4 The impacts of silver nanoparticles (AgNPs) on plant growth

AgNPs have shown a greater efficacy in enhancing seed germination. Several previous studies have shown their applications in plant growth parameters. The proper AgNPs concentrations have remarkably improved the growth and yield of crops. Many studies confirmed that AgNPs positively improved crops with less or no phototoxic effect on plant growth. In contrast, negative effects were also reported on several plant species.

### 4.1 Positive effects on plant growth

AgNPs are used as antiviral, antibacterial, and antifungal agents in agriculture application. It preserves the shelf life of fruits, foliage, flowers, and vegetables and stimulates plant growth and metabolism. The plant responses to the AgNPs applications are diverse, depending on the exposure time, types of plant species, composition, particle size, concentration, functionalization, and many other factors. In a study, AgNPs of different concentrations were used at 0, 5, 10, and 20 mg/L. The root development as novel bio stimulants enhanced upon their application at 5 mg/L. In addition, they also improved nitrogen (N), phosphorous (P), and potassium (K) concentration in leaves, which is beneficial in enhancing seedling performance during the primary developmental stages [58]. Exogenous application of AgNPs diversely affected plant growth. The treatment of *Oryza sativa* at 30 μg/mL of AgNPs enhanced root growth, while 60 μg/mL stopped root growth [59]. An experimental study investigated the AgNPs effect on mean germination time, rate, percentage, root length, and dry/fresh weight of seedlings for the three spices. AgNPs at different concentrations such as 0.05, 0.1, 0.5, 1, 1.5, 2, and 2.5 mg/L were used to investigate the seed germination stage. Three spices have shown varying dosage responses to AgNPs on measured growth characters and germination percentage. Germination rate values of three plants have improved in response to AgNPs [48]. Using the seed growth-mediated technique AgNPs with a size of 13 nm were synthesized on Ag seeds with a size of around 6 nm. The application of AgNPs with 50 mg/L enhanced the highest chlorophyll accumulation and lowest carotenoid accumulation in the leaves, lower GPOX activity, and fewer anthocyanins and polyphenols with 100 mg/L. AgNPs-derived seedlings showed smaller anthocyanins, carotenoids, and chlorophyll contents in kale. Still, higher GPOX activity was characterized by higher dry shoot and fresh weights and higher heterogeneous biometric parameters of the roots [[60], [61]]. Trisodium citrate salt based synthesize AgNPs (9 × 10^−4^ M) were sprayed at 0, 50, and 75 ppm on wheat crops of different species: *Vigna sinensis*, *Triticum aestivum* and Brassica *juncea.* The application of AgNPs at 50 mg/L enhanced wheat (cowpea) shoots' dry weight and length. The shoot enhancement was reported at 75 ppm in Brassica. AgNPs were synthesized and contaminated soil with AgNPs to check their impact on different plants, such as *Lactuca sativa* (lettuce), and *Avena Raphanus sativus* (radish) and *byzantina* (oat). It produced a higher percentage of dry mass and root growth [[30], [31], [62]]. Foliar AgNPs treatment at 20, 40, and 60 mg/L has enhanced the growth parameters of the fenugreek plant (shoot dry weight, number of plant/leaves, and shoot length). Other biochemical aspects were improved, such as the concentration of indole acetic acid (IAA) and photosynthetic pigments such as the concentration of indole acetic acid (IAA) and photosynthetic pigment such as chlorophyll *a*, chlorophyll *b* and carotenoids. It also improved the yield quantity (number of seeds/pod, seed index weight of seeds/plant, and number of pods/plant), antioxidant activity, and quality (carbohydrate%, protein%, tannins, flavonoids and phenolics contents) of the yielded seeds [10]. The application of AgNPs at different concentrations such as 0, 25, 50, 100, 200, and 400 ppm enhanced the antioxidant status and growth of 7-day-old *Brassica juncea* seedlings. AgNPs affected the vigor index, shoot length, root length, and fresh weight seedlings. Root length and vigor index were increased to 326% and 133% of the treated seedlings. There was evidence of increased chlorophyll concentration and photosynthetic quantum efficiency in leaves of treated seedlings [63]. The chemically synthesized AgNPs (100 nm) based on polyvinyl pyrrolidone (PVP), and biologically synthesized using leaves of *Ricinus communis* L were used to evaluate the growth of water plant. AgNPs (biologically and chemically) application at 1, 10 and 100 mg/L on the growth and physiology of an aquatic plant water *hyacinth - Eichhornia crassipes* (Mart) Solms. Chemically synthesized AgNPs have been found to reduce the growth of hyacinth, whereas biological AgNPs have not demonstrated this effect. AgNPs (10–30 nm) of 15 ml were daily supplied at different concentrations: 20, 40, 60, 80 and 100 ppm respectively. The results indicated that less concentration induced plantlets while higher concentration caused inhibition [33,34]. In a study, lotus was examined in the crops after 4 times treatments with AgNPs (4 mg/L): control, soil treatment before 5-days of planting with 4 mg/L (T1), treatments before planting with 4 mg/L (T2), and combined periodic foliar application, plant and soil treatments at 4 mg/L (T3). The different methods of AgNPs application induced leaf diameter, plant height, dry and fresh leaves weight, and some biochemical aspects [[64], [65], [66]]. AgNPs were synthesized using leaves extract of *Coriandrum sativum* and tested on *Lupinus termis* L at 0, 0.1, 0.3, and 0.5 mg/L for 10 days. The result evidenced that less concentration enhanced growth, while higher reduced the growth [67]. AgNPs at 0-, 10-, 20-, and 30-mM increased plant growth and maintained the ionic balance of cells (Na^+^, K^+^, and Na^+^/K^+^ ratio) by decreasing the uptake of Na and Cl and oxidative stress by salt-stressed plants. It also induced catalase activities, superoxide dismutase, and reduced peroxidase activity. Whereas the reduction of MDA and H_2_O_2_ contents was reported in plants under salt stress. The application of AgNPs in a dose-additive manner enhanced the yield, height, and photosynthesis pathway of the salt-stressed plants [68]. *Moringa oleifera* extract was employed in the production of AgNPs. The AgNPs application at 25, 50, 75, and 100 mg/L protected against heat stress, increased shoot length (22.2 and 26.1%), root length (5 and 5.4%), plant dry weight (0.36 and 0.60%), fresh plant weight (1.3 and 2%), and root number (6.6 and 7.5%) at 50 and 75 mg/L. AgNPs application at 50 and 75 mg/L have significantly induced leaf number (4 and 4.8%), leaf area (18.3 and 33.8%), dry leaf weight (0.06 and 0.18%), and fresh leaf weight (0.09 and 0.15 in over the respective value of control under heat stress [69]. The extract of pistachio seed coat waste was utilized to synthesize AgNPs. The solution of AgNPs at 75 mg/L was sprayed on egg plants which enhanced carotenoid/chlorophyll contents and plant growth. The addition of fly ash induced the plant growth. AgNPs were sprayed at 75 ppm of concentration on 20% fly ash amended soil which remarkably improved plant growth [70].

### 4.2 Negative effects on plant growth

The toxicity of AgNPs to plants in terrestrial ecosystems, notably crops, is concentration and size-dependent. It reduced germination and growth of seed and affected roots/shoots length and mass. Its accumulation in leaves and roots activates the defense mechanism at cellular/tissue levels and modifies proteomic expression, antioxidant activities, and metabolism. Botanical changes such as generation of reactive oxygen species, total chlorophyll, superoxide dismutase activities, increased level of H_2_O_2_, glutathione, carotenoid, ascorbate, proline contents, etc., that were either increased or decreased after exposure to AgNPs. These processes caused the suppression of photosynthesis/transition, irregular morphological modifications, and other symptoms [71]. AgNPs were synthesized using *Aloe vera* extract. AgNPs and AgNO_3_ at 40, 60-, 80-, 100-, and 120-mM reduced *Brassica* seedling growth due to a higher accumulation of AgNPs and AgNO_3_ and severely inhibited photosynthesis. Its interaction with the metabolism and growth of mustard seedlings has imposed mild stress conditions [72]. The treatment of AgNPs at 20, 200, and 2000 mg/kg on wheat (*T. aestivum)* resulted in less plant weight, shorter plant height, and lower biomass [73]. AgNPs were synthesized using the citrate reduction method and were stabilized with chitosan. A varied AgNPs concentration of 12.5, 25, 50, and 100 ppm was given to the *Lactuca sativa* L. (Asteraceae) seeds to assess the possible danger of AgNPs utilizing percentage of germinated seed and root morphological alteration as toxicity parameters. At a concentration of 100 ppm, there was a reduction in root growth compared to the positive control (distilled water) [74]. Citrate-coated AgNPs were synthesized and purified. The treatment of AgNPs at 1 mg/L reduced the biomass and height of *Capsicum annuum* [75]. The exogenous AgNPs application at 0.2 μg/L inhibited root hair development in *Arabidopsis thaliana*. AgNPs were synthesized and stabilized using PVP. The AgNPs at 100–900 μg/kg were used in the soil. The germination rate at 800 μg/kg in soil was reduced *Vicia faba, R. leguminosarum bv. viciae* growth and *G. aggregatum* [45,46]. The phototoxicity of AgNPs (10 nm) was evaluated to study hydroponic plant growth. Similarly, a higher concentration of Ag ions (2.5 mg/kg) decreased plant growth to the same extent as the AgNPs [[76], [77], [78]].

## 5 Applications of AgNPs in agriculture sector

Nanotechnology is the most advanced field in agriculture and is tremendous scientific interest globally. The definition of nanotechnology states that the nanoscale is the smallest particle ranging from 1 to 100 nm in size. The significance of metallic nanoparticles is very high in several fields. AgNPs have been a primary focus for researchers due to their unique biological, chemical, and physical properties. AgNPs are crucial in the bio-system that are well studied and utilized. It has a broad spectrum of anti-microbial activities and strong bactericidal/inhibitory effects. AgNPs are the most important in agriculture that involves in enhancing seeds germination and plant growth. Several applications of AgNPs including: Nano-pesticides, Nano-fertilizers, food packaging, pest management, crops, and food protection (nano-antibacterial, nano-antifungal, and nano-antiviral agents).

### 5.1 AgNPs as nano-pesticides

**Fig. 1:**
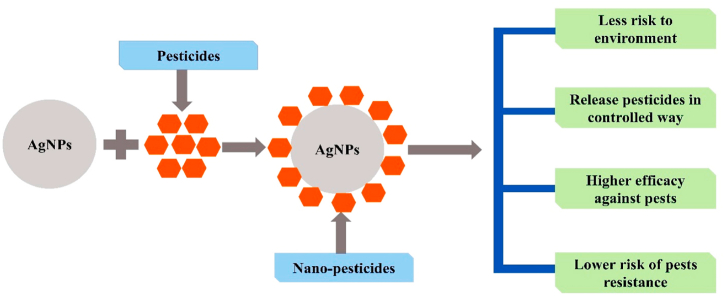
Silver nanoparticles incorporate with pesticides and form AgNPs based nano fertilizers that kill and reduce pest resistance.

Pests are emerging threat to the agricultural sector, affecting crop yield, reducing crop quality, and applying synthetic pesticides to the soil and plants, negatively impacting the environment. Nano pesticide is a novel tool for controlling and managing pests with agricultural significance. AgNPs act as pesticides and reduce the pest burden on the crop. The most critical importance of nanocides (nanotechnology-based pesticides) is their eco-friendly relationship to the environment and minimal effect on non-targeted insects. It provides nutritional enrichment to the plants and protects against pests. Thus, constant usage of chemical fertilizer is reducing in conventional farming. It also eradicates harmful microorganisms in hydroponics systems and soil. The foliar spray application is beneficial in inhibiting rot, moulds, fungi, and numerous other microbial-associated plant diseases [79]. AgNPs were biologically synthesized from *Ulva lactuca* that have ability to reduce silver nitrate into AgNPs, and the formation of brown color confirms their presence in the solution. The anti-microbial activity of biologically synthesized pesticides showed their fight against fungi and bacteria, causing various crop diseases [80]. AgNPs of 50–100 nm was synthesized using *Solanum lycopersicum*. AgNPs application concentrations at 200, 300, 400, and 500 ppm were applied to *M. rosae,* and distilled water was used as a control. The mortality rate of AgNPs showed optimum efficacy at 500 ppm concentration. The mortality rate was associated with higher concentrations [81]. In a study, three types of AgNPs (10 nm) were used to check larvicidal potential on the survival of the sixteenth larval stage *Tenebrio molitor*. AgNPs and silicon dioxide microparticles revealed more than 70% insecticidal effects on larval viability [82]. PVP-coated-AgNPs of different concentrations were used against two lepidopteran pests of the *Ricinus communis* L. (castor plant), namely castor semilooper, *Achaea janata* L. Larvae and Asian armyworm, *Spodoptera litura F*. Electron microscopic studies have demonstrated that AgNPs easily penetrate the plant, insect cell, nucleus, or mitochondria. It can also be employed as a pesticide for targeted delivery (Fig. 1) [83].

### 5.2 AgNPs as nano-fertilizers

**Fig. 2:**
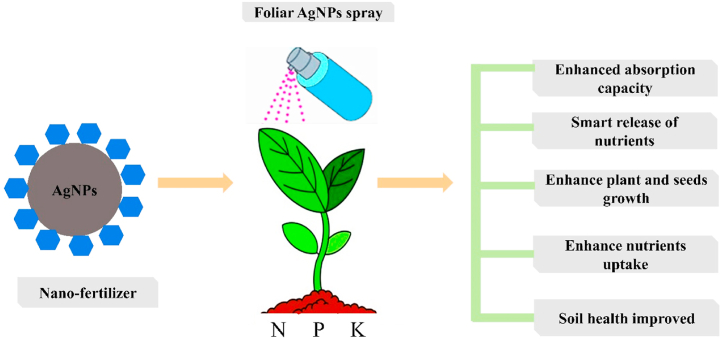
The foliar spray of AgNPs as nano-fertilizers enhances nutrient uptake and plant growth.

The utilization of AgNPs as a nano fertilizer are considered the most effective approach for adequately management of plant nutrient supply. Excessive fertilizers were lost in the field, that adversely affected the environment. Nanoparticles bind to the fertilizers and release the proper number of fertilizers to the plants. Thus, in this way, the excess usage of fertilizer is reduced in the field. AgNPs have demonstrated importance across various aspects of agriculture. The synthesis of nano fertilizers from extract of onions were found effective for tomato and brinjal plants. AgNPs act as nano fertilizers that slowly and effectively release nutrients to prevent the loss of nutrients. It helps the plant to enhance nutrients absorption from the soil [84]. The nano-biofertilizer reduces farm management costs, environmental pollution, and unnecessary chemical fertilizers in the field (Fig. 2) [85]. AgNPs were synthesized using the entomopathogenic fungi, *Beauveria bassiana* (Bb), and *Metarhizium brunneum* (Mb). The efficacy of Mb-synthesized and Bb-synthesized AgNPs were tested against *Tetranychus urticae Koch* (Acari: Tetranychidae); a two-spotted spider mite. The results revealed that both treatments were significantly virulent toward the newly emerged adult females of *T. urticae* [86]. Combining AgNPs with magnetic field showed the highest fruit yield (16.420-ton/ha) in muskmelon (*Cucumis melo* L.). The treatment of AgNPs showed 150% more fruit yield than the control [87]. In a study, AgNPs at 0, 25, 50, 75, 100, 125, and 150 ppm were used for various seedling varieties. The growth, yield, quality of NPK uptake, and nutrient efficiency was improved in wheat. Remarkable leaf growth and yield were observed at 25 ppm, while 75 ppm showed less yield of the wheat [88]. The aqueous root bark extract of *Berberis lycium Royle* was used to synthesize AgNPs. The impacts of AgNPs at 0, 30, 60, and 90 ppm were used to observe carbohydrate and protein contents of *Pisum sativum* L. seed. The highest carbohydrate and protein contents were recorded in harvested seeds [89].

### 5.3 Utilization of AgNPs in insects/pest management

The nanoparticles incorporate into insects and rapidly control their progression. AgNPs is an effective agent for pest management with numerous properties including non-toxic, safe and improved pest control method. Nano-based pesticides efficiently provide a high and proper dose to the target plants [90]. The nanoscale application of agrochemicals has changed the traditional agro-practices in nano-formulations, nano-pesticides, nano-sensors, and nano-fertilizers [91]. Inorganic-based nano pesticides have been tested for stored-based insect/pest management. The plant-based nanoparticles are significant in controlling pests in stored grains. It is ecofriendly and easy way to develop as compared to chemical-based synthesis of the nano-pesticides [92]. AgNPs are used as carriers to deliver agrochemicals to the targeted site [93]. The mortality tests, repellent activity, antifeedant tests and ovipositional deterrency confirmed that the malathion-based AgNPs determine maximum pesticidal activity against *Tribolium castaneum*. *Tribolium castaneum* is a significant beetle that infests stored grains and shows resistance to commercial synthetic insecticides. AgNPs and malathion in different concentrations were added to the disk at 50 ppm, 75 ppm, and 100 ppm. The mortality of AgNPs and malathion at 75 ppm was 75% and 95%, respectively. Therefore, AgNPs have proven effective against pests and beetles [94]. AgNPs also act as bactericidal and larvicidal. The marine bacterium *Shewanella algae bangaramma* were used to synthesize AgNPs. The maximum LC_50_ and LC_90_ values with a 95% confidential limit (4.529 mg/mL (2.478–5.911), 9.580 mg/mL (7.528–14.541) were reported with III- instar larvae Burmeister (*Lepidiota mansueta*). The mortality of the larvae was enormously enhanced in exposed groups at all concentrations (p < 0.0001). The marine bacteria-mediated synthesis of AgNPs in a culture medium provides efficient antifouling and larvicidal activities [95]. *Bacillus thuringiensis* kurstaki (Btk), an entomopathogenic bacteria, was used to produce Btk-AgNPs. The insecticidal activity of *Btk*-based synthesized AgNPs was tested against larvae of *Agrotis ipsilon* (Hufnagel) and *A. ipsilon* (Hufnagel). The application of either *Btk*-synthesized AgNPs using *Bt* pellet or *Btk*-synthesized AgNPs made with *Bt* supernatant were highly virulent toward larvae of *T. ni* compared to *Agrotis ipsilon* [96]. AgNPs of 3–25 nm size were synthesized using entomopathogenic fungus *B. bassiana* from both isolates. There were two groups, i.e., 50% and 51–100% of 20 isolates. The maximum mortality was 64% in 90 isolates. Its potency against mustard aphid (*L. erysimi*) showed the potential of AgNPs to manage insects in agriculture [97]. AgNPs were synthesized using *Leonotis nepetifolia.* AgNPs exhibited possible antifeedant activity against the larvae of *S. litura* (78.77%) and *H. armigera* (82.16%). *S. litura* and *H. armigera.* AgNPs also showed a high larval mortality rate and maximum pupal mortality rate (78.49 and 72.70%) and (84.66 and 77.44%), respectively. The biosynthesized AgNPs were tested on mosquito larvae and recorded LC_50_ values were *C. quinquefasciatus* 35.48 ppm and *A. aegypti* 47.44 ppm. The production of AgNPs using *L. nepetifolia* is an environmentally favorable method for management of insect pests. The histological examinations confirmed that increasing nanomaterial resulted in severe tissue damage in the goblet and epithelial cells of the larval midgut region of *H. armigera*, *S. litura*, *C. quinquefasciatus,* and *A. aegypti* [98].

### 5.4 Utilization of AgNPs in food packaging

Nanotechnology used the tiniest particles, measuring one billionth of a meter which have already been used in food packaging, functional food ingredients, and food supplements. One of the fields where nanotechnology is predicted to play a key role in the future is food technology. Food additives (nano inside) and food packaging are the two most common forms of nanofood applications (nano outside). For example, nanoscale food additives can be used to influence product shelf life, texture, flavor, nutritional content, identify food pathogens, and serve as food quality indicators. Nanotechnologies are regarded to be effective in the context of food packaging for enhancing product shelf life, detecting rotten components, and generally improving product quality, such as by minimizing gas flow over product packaging [99]. The essential branch of agriculture in preserving agricultural products is post-harvest management. Nanotechnological packaging applications are; reduced hydrophilic characteristics, better biodegradability, and physical-chemical properties [100]. Active packages promote a new generation of food packaging obtained by incorporating metallic nanoparticles into polymer films. AgNPs combine with edible and non-degradable polymers for active food packaging [101]. Incorporating AgNPs with hydroxypropyl methylcellulose (HPMC) matrix applications provides the best packaging materials of food. Mechanical analyses and water vapor barrier properties of the HPMC/AgNPs nanocomposites showed the best result for films containing smaller size (41 nm) AgNPs. HPMC/AgNPs nanocomposites are used in active anti-microbial internal coating in food packages [102]. A solution-based casting method was used to fabricate a hybrid nanocomposite film that consists of AgNPs, polyethylene glycol, gelatin and chitosan.

Several film series were synthesized having different concentrations of AgNPs and chitosan. AgNPs were used to increase mechanical qualities while decreasing light transmission in the visible light spectrum. By using the hybrid film for packaging red grapes, the fruit shelf life was extended further two weeks [103]. A reduction method utilized an agent NaBH_4_ to synthesize CNF/AgNPs composite, which is used as an anti-microbial material in active food packaging systems [104]. Low-density polyethylene films (LDPE) with AgNPs have the potential to greatly improve the safety, and quality of packaged food. In addition, it improve the antimicrobial properties and extending its shelf life [105]. CNC/AgNPs and CMC/AgNPs were developed for paper coating. This packaging of strawberries extends shelf-life to 7 days under ambient conditions [106]. Using rapid biological methods AgNPs were synthesized by utilizing leaf broth of *Capparis zeylanica*, that enhanced the stability of the PVA/PEG polymer. The thin-film composite displayed outstanding characteristics in every aspect, enabling it for food packaging and biomedical applications [107]. AgNPs combination with low-density polyethylene films at 1.50, 3.75, 7.50, 15.00, 30.00, 60.00, and 75.00 μg/mL have shown anti-microbial properties against microorganisms. It would be the most effective tool for improving food safety and quality [108].

### 5.5 Applications of AgNPs in crops and foods protection

Healthy plants are most important for animal and people health, but they often receive less emphasis and attention in health literature. Plants are the most important source of nutrition for cattle and provide more than 80% of human food. Pest infestation and plant diseases threaten plant availability and safety of human and animal consumption. Globally, losses of critical staple crops make up 30%, and billions of dollars were lost in the production of food [109]. Nanotechnology is the most effective and safe technique, providing healthier food and enhancing precision farming [110]. They can eliminate target microbes from hydroponics, soil, and plants by protecting plants in various ways, such as acting as nano-antiviral, antibacterial, and nano-antifungal.

#### 5.5.1 AgNPs as nano-antibacterial agents

**Fig. 3:**
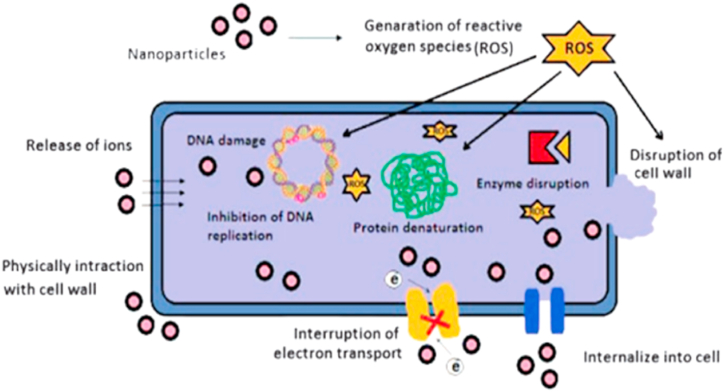
Antibacterial mechanism of AgNPs.

AgNPs have versatile applications such as biosensors, antioxidants, and heavy metal detection [111]. They have unique physicochemical properties, including high surface reactivity, binding capabilities with biological molecules, large surface area to volume ratio, ease of characterization, and synthesis. AgNPs can also enhance the expression of genes in redox processes. These properties allow us to use AgNPs as anti-microbial agents against the pathogenic disease of plants and other foodborne infections [112]. AgNPs affect bacterial communities in numerous agricultural soil, either harmful or advantageous to plants and the environment [113]. The antiviral and antibacterial activities of the silver ions (Ag^+^) could be increased to one thousand-fold when the carbonate ions concentration is less than drinking water norms [114]. AgNPs were synthesized using *S. orvum* based fruit extracts. The GO-Ag nanocomposite was prepared and applied to both *S. aureus* ATCC 6538 and *E. coli* ATCC 25922 by plate count and disk diffusion methods, which showed excessive antibacterial activity [115]. AgNPs were synthesized using *Ziziphus joazeiro* leaf extracts. Particles with less aggregation degree and smaller size were obtained in neutral conditions (pH-7) and exhibited antibacterial activities against *S. aureus* and *E. coli* [116]. AgNPs were synthesized using fruit extract of *Solanum torvum* which showed minimum inhibitory concentrations against bacterial plant pathogens *Xanthomonas axonopodis* pv. *punicae* (6.25 μg/mL) and *Ralstonia solanacearum* (12.5 μg/mL). The inhibition zones *in vitro* disk-diffusion assay is *R. solanacearum* (11.4 ± 1 mm) and *X. axonopodis* pv. *punicae* (18.1 ± 1 mm) after AgNPs application at 50 μg/mL [117]. Silver nitrate (AgNO_3_), sodium borohydride (NaBH_4_) and 1% sodium citrate dihydrate were used to synthesize AgNPs. The phytopathogenic fungi (*Ralstonia solanacearum*) cause severe bacterial wilt in tobacco. It has determined the bactericidal and bacteriostatic activity of surfactant-stabilized and pure AgNPs. Surfactants had varying effects on the antibacterial activity of AgNPs against *R. solanacearum* [118]. Fig. 3 illustrates the antibacterial mechanism of AgNPs.

#### 5.5.2 AgNPs as nano-antifungal agents

AgNPs are efficient and fast-acting fungicides against broad-spectrum fungal strain like *Saccharomyces*, *Aspergillus* and *Candida*. The extracted AgNPs using *bovine mastitis* with a diameter 13.5 ± 2.6 nm are considered most potent against yeast [119]. An efficient concentration of AgNPs inhibits 50% (EC50) of colony formation in *B. sorokiniana* than for *M. grisea*. Nanoparticles and silver ions stimulate the spores colony formation and plant-pathogenic fungi-related disease [120]. Stock AgNPs solution at 4000 ppm concentration was obtained from Nanocid Company. AgNPs were used with potato dextrose agar medium to grow fungal isolates *in vitro*. AgNPs at 6, 8, 10, 12, 14, and 16 ppm were used on five phytopathogenic fungi. The radial fungal growth was noted after 1, 2, 3, 5, and 10 days, and further calculated inhibition rates of mycelial growth. The experimental greenhouse results has revealed that treatments with fungicide and AgNPs provide a better yield than the positive control [121]. *Cassia roxburghii* aqueous leaf extract was used to synthesize AgNPs. The phytogenic AgNPs were utilized to combat several plant pathogenic fungus, which showed antifungal activity against *Curvularia* sp.*, Fusarium oxysporum,* and *Rhizoctonia solani* [122]. Aldehyde-modified sodium alginate (ASA) was used to synthesize AgNPs. The antifungal activity of AgNPs primarily alters the cell membrane permeability, affects the soluble protein synthesis, destroys DNA structure, and inhibits DNA replication. It has not shown any inhibition in *N. benthamiana* and rice seed germination [123]. AgNPs at temperature of 95 °C were synthesized by using a chitosan-reducing agent. The chitosan-AgNPs based-composite showed remarkably higher antifungal activity against *Colletotrichum gloeosporioides* [124]. AgNPs were synthesized by using leaf extract of *Amaranthus retroflexus*. AgNPs at 50, 100, 200, and 400 μg/mL were applied to the human pathogenic fungi, mushroom, and plant growth. The minimum inhibitory concentrations (MIC_50_) of 50% were observed against *Fusarium oxysporum* (328.05 ± 13.29)*, Alternaria alternata* (337.09 ± 19.72), and *Macrophomina phaseolina* (159.80 ± 14.49) μg/mL [125]. AgNPs (WA-CV-WA13B, WA-AT-WB13R, and WA-PR-WB13R) were obtained from Bio Plus Co. (Pohang, Korea). Various concentrations (10, 25, 50, and 100 ppm) were used against eighteen plant pathogenic fungi. Maximum inhibition was recorded using CV-WB13R AgNPs and at 100pm treatments [126]. AgNPs were synthesized chemically (AgNO_3_) and biologically (fresh plant leaves). The anti-microbial potential of AgNPs was evaluated, which showed different inhibition against three plant pathogenic fungi; *Alternaria solani* and *Fusarium* spp. (100%) and *Corynespora cassiicola* (85%) at 100 ppm [127].

#### 5.5.3 AgNPs as nano-antiviral agents

**Fig. 4:**
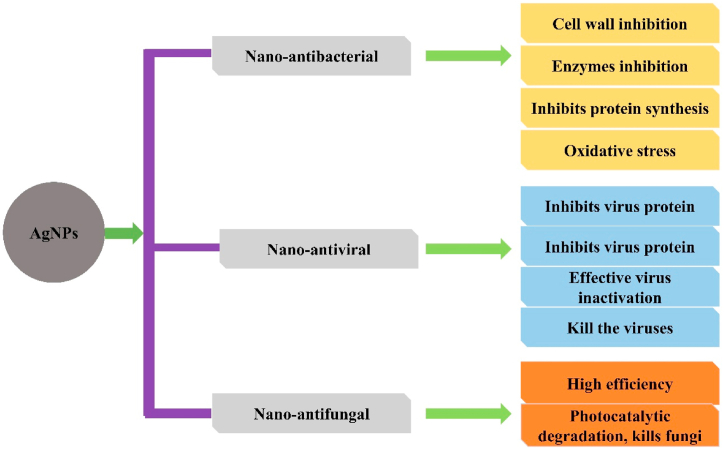
AgNPs act as nano-antibacterial, nano-antiviral, and nano-antifungal agents.

Several studies exhibited that metal-based nanoparticles such as Ag, Au, Cu, Fe, etc., showed broad-spectrum antiviral activities, which enable them to use broadly in the disinfection of air/water and medicines. The metal-based nanoparticles that inactivate the virus may have an essential role before and after the entry of the virus into the host cells. The virus absorption occurs on the surface of metal and their glycoproteins moiety interact with the metal particles by making them inactive. It may also penetrate cells and interact with viral nucleic acids, inhibiting their antiviral potential [[128], [129], [130], [131]]. The adsorption effect of AgNPs is high in the virus that interacts either with the surface protein or viral envelope, affecting the virus and cell receptor interaction and preventing the virus attack on the cell. The combination of AgNPs with the virus causes the release of Ag ions. It inactivates them by reacting with a viral protein or viral nucleic acids to inhibit the replication of viruses. AgNPs surface activates the oxygen and synthesizes Reactive Oxygen Species (ROS). It oxidizes the virus by damaging its structure (Fig. 4) [[98], [99], [100]]. The bacterial strains such as *Bacillus persicus*, *Bacillus pumilus* and *Bacillus* licheniformis were used to synthesize AgNPs (77–92 nm). *B. licheniformis* has been demonstrated to be effective against both the Bean Yellow Mosaic Virus and as well as human pathogen [132]. By reducing silver nitrate; AgNPs were synthesized. AgNPs have been conducted on viricidal activities and deactivated Bean yellow mosaic virus (BYMV). The disease was inhibited at 100 mg/L, while the AgNPs ultimately arrested the virus infection at 200 mg/L [133]. AgNPs were obtained from Sigma company in a liquid form. AgNPs were sprayed on banana plants infected with BBTV *(Banana bunchy top virus)* at three different concentrations (40, 50, and 60 ppm). The infection rate was 36%, and the genomic DNA level changed after 50 ppm of treatment. The result showed that AgNPs could be used in the future against plant viruses [134]. Silver nitrate was used as reducing agent to synthesize AgNPs. Five different concentrations of antiviral drug (25, 50, 100, 150, and 200 ppm) were used to diminish TSWV infection on *Ch. amaranticolor* and potato plants. All concentrations showed inhibitory effects, but strong inhibitory effects (90.4%) at 200 ppm were recorded against Tomato spotted wilt virus. However, a similar concentration also showed an inhibitory effect of 87.5% against Tomato spotted wilt virus (TSWV) systemically infected potato seedlings [135]. AgNPs were synthesized and bio-fabricated using *Pseudomonas fluorescens* CZ strain fermentative broth. AgNPs of 100 μl was applied to the leaf for 24 h and mixed with tobacco mosaic virus (TMV). AgNPs were shown to be highly effective against the infection of RNA-based plant viruses [136]. AgNPs were obtained from Sigma in a liquid form. AgNPs were sprayed at concentrations such as 50, 60, and 70 ppm for 7 days period. The results indicated that 50 ppm treatment reduced the relative concentration of both viruses and the severity of diseases [137].

## 6 Conclusions

The current review findings depicted that AgNPs have a crucial role in increasing agricultural productivity, seeds and growth of the plant. The most convenient application of AgNPs is that they act like fertilizers that reduce nutrient loss and control plant infections, similar to pesticides, which might enhance plant productivity. They also significantly improve soil quality by improving the nutrient supply and removing soil containments. AgNPs also enhance the water supply and provide essential nutrients properly to the plant. AgNPs have diverse applications in different fields, especially in agriculture. They also act as nano-fertilizers and nano-pesticides, reducing salt stress and improving the plant’s production. AgNPs protect plants against harmful bacteria, fungi, and viruses by acting as nano-bacterial, nan-fungal, and nano-antiviral. Similarly, they also provide support for food packaging, which protects food from gases (CO_2_, O_2_), light, dust, moisture, and pathogens. Therefore, it can be concluded that AgNPs should be utilized in agriculture sector to promote plant growth and increase productivity of crops.

## 7 Future perspective

Nanotechnology is a new field of study that investigates the unique physicochemical features of the nanoparticles and their applications in various fields such as agriculture. AgNPs are used widely for their applications in agriculture sector. They promote plant growth as they are used as nano-fertilizers, also these nanoparticles are utilized as nano-pesticides by controlling different insects and pests that attack on plants. Furthermore, they are also used as nano-bacterial, nan-fungal, and nano-antiviral agents against various microbes that cause diseases in plants. In agriculture sector low yield and crops diseases are significant issues which are under consideration, therefore researchers should work in this field to explore the efficacy of AgNPs in plant growth and agriculture. The impacts of ecofriendly and bio-based AgNPs should be tested on different plants and agriculture crops. Further research work is needed to overcome the problems of low yield and control plants diseases by the utilization of biosynthesized AgNPs and other less toxic metals.

## Author contribution statement

All authors listed have significantly contributed to the development and the writing of this article.

## Data availability statement

No data was used for the research described in the article.

## Additional information

No additional information is available for this paper.

## Declaration of competing interest

The authors declare that they have no known competing financial interests or personal relationships that could have appeared to influence the work reported in this paper

## Abbreviations

AgNPs: Silver nanoparticles
Btk: Bacillus thuringiensis kurstaki
BYMV: Bean yellow mosaic virus
BBTV: Banana bunchy top virus
CNF: Cellulose nanofibril
DNA: Deoxyribonucleic acid
E.N.M. s: Engineered nanomaterials
Ga: Gum Arabic
gp: Germination percentage
GO: Graphene oxide
GPOX: Glutathione peroxidase
IAA: Indole acetic acid
LC: Lethal concentration
mL: Milliliter
mg: Milligram
nm: Nanometer
GNPs: Green Nanoparticles
NPK: Nitrogen Phosphorous Potassium
Ppm: Parts per million
PVP: Polyvinylpyrrolidone
ROS: Reactive oxygen species
SVI: Seedling vigor index
TSWV: Tomato spotted wilt virus
mg: Microgram
VI: Vigour index
LDPE: low-density polyethylene
